# Surface Morphology and Subsurface Microstructure Evolution When Form Grinding 20Cr2Ni4A Alloys

**DOI:** 10.3390/ma16010425

**Published:** 2023-01-02

**Authors:** Xiaodong Zhang, Xiaoyang Jiang, Maojun Li, Pan Gong

**Affiliations:** 1 State Key Laboratory of Advanced Design and Manufacture for Vehicle Body, Hunan University, Changsha 410082, China; 2 State Key Laboratory of Materials Processing and Die & Mold Technology, School of Materials Science and Engineering, Huazhong University of Science and Technology, Wuhan 430074, China

**Keywords:** microstructure evolution, surface morphology, form grinding, grinding defects

## Abstract

20Cr2Ni4A alloy is widely used in the manufacturing of heavy-duty gears, although limited information about its machinability during the form-grinding process has been reported. In this work, form-grinding trials on transmission gears of 20Cr2Ni4A alloy under various parameters were conducted. Surface morphology of the gear tooth, surface roughness distribution and microstructure evolution of the machined surface layer were comprehensively studied, and the influence of grinding parameters on grinding performance was investigated. The formation mechanisms of surface/subsurface defects during the form-grinding process, including plastic flow, deep grooves, successive crushing zone, adhesive chips and cavities, were analyzed. Results showed that the change in contact conditions between the grinding wheel and tooth surface led to the decrease in the surface roughness from tooth tip to root. Mechanical force and grinding heat promoted the deformation and refinement of the microstructure within the machined surface layer. With the increase in cutting depth and feed speed, the deformation ratio of the microstructure increased, which was also consistent with the variation trend in the form-grinding temperature.

## 1. Introduction

The transmission gear is a key component in a vehicle, and its reliability affects the safety of the transmission system, as well as the entire mechanical system [1]. The 20Cr2Ni4A alloy is widely used in gear manufacturing because of its high strength, toughness, hardness and relatively low cost [2]. However, the excellent mechanical properties also make 20Cr2Ni4A steel a typical, difficult-to-cut material. Due to the important function of the transmission gear in mechanical systems, high surface quality is essential for machining components using the 20Cr2Ni4A alloy. The form-grinding process of gears has the advantages of a high material removal rate, profile accuracy and processing efficiency. It is commonly used as the final procedure in the gear manufacturing process chain to eliminate deformations in the gear blanks introduced by heat treatment and to improve the tooth surface quality [3]. After the grinding process, the surface integrity of the gear tooth, such as surface morphology, roughness and microstructure, greatly affects the gear performance and determines the reliability of gear products [4,5].

The grinding process requires a high energy input, and most of the energy is ultimately converted into heat within the workpiece, chips, grinding wheel and coolant. The grinding heat is difficult to transfer uniformly within the workpiece in a short period. Thus, the temperature in the grinding contact zone increases sharply, and the temperature gradient becomes large, which may generate grinding burns and surface cracks and deteriorate the surface integrity [6]. The typical grinding parameters are wheel speed, cutting depth and feed rate. Previous studies have indicated that grinding parameters had significant effects on grinding temperature. Su et al. [7] studied the influence of process parameters on grinding temperature during the gear form-grinding process. The increase in cutting depth and wheel speed increased the heat-flux density in the grinding zone, resulting in an increase in the grinding temperature. Increasing the feed rate, on the contrary, reduced the loading time of the heat source and decreased the grinding temperature. Yi et al. [8] form-ground Gr15 gears and found that the contact geometry on the tooth surface varied with the rolling angle of the involute profile, and the local grinding parameters were variable at different contact points. As the maximum local normal grinding depth, tangential grinding force and the minimum contact arc length were recorded at the tooth tip. The maximum grinding temperature was generated near the tooth tip during gear grinding.

During the grinding process, with the high-speed mutual movement between the grinding wheel and the workpiece, the machined surface is scratched, ploughed and cut by abrasive grits. Severe plastic deformation of the workpiece material occurs along the cutting path of the abrasive grits. Under the action of high shear stress, some of the materials in front of the abrasive particles are separated from the workpiece and form chips, while others flow to both sides of the cutting path and pile up [9], which greatly affect the surface morphology of the gear tooth. In view of the intensive interaction between the grinding wheel and the workpiece, defects such as abrasive chips, deep grooves and plastic flow often occur on the machined surface after the form-grinding process. Yang et al. [10] ground 20CrMnTi gears and found that, when the cutting depth was 0.1 mm, small areas of coatings and sporadic adhesive chips scattered on the tooth surface. With the cutting depth increased to 0.15 mm, a large number of adhesive chips and deep grooves were observed. When the cutting depth increased to 0.2 mm, microcracks appeared on the machined surface. In addition, it was also reported that an increase in the feed rate increased the degree of plastic flow and the depth of scratches on the machined surface [11]. Ma et al. [12] investigated the influence of grinding parameters on the surface roughness of the gears using 45# steel. With the increase in feed rate, the residence time of the abrasive grits on the tooth surface was reduced, and the material removal during the gear grinding was not sufficient. As a result, the tooth surface roughness increased owing to an increase in the average undeformed chip thickness. As the cutting depth increased, the grinding width and contact arc length between the wheel and tooth surface increased. Thus, the unevenness of the tooth surface material removal was increased, resulting in an increase in tooth surface roughness. Zhou et al. [13] studied the distribution of tooth surface roughness during gear form grinding. The local linear speed of the grinding wheel became larger at the position closer to the tooth root, and the grinding traces were more likely to overlap with each other. 

Scratching, ploughing and cutting occurring at the interface between the grinding wheel and machined surface are considered to induce the coupling effect of mechanical force and grinding heat, and change the microstructure of the workpiece surface layer [14]. In the grinding process, the loading of mechanical force usually causes severe plastic deformation within the surface layer and introduces a large number of dislocation structures. Large grains in the surface layer are compressed and crushed. The average size of the grains becomes smaller when approaching the machined surface [15]. When the maximum grinding temperature is above the equilibrium phase-transition temperature of the workpiece material, the surface layer, in the a of rapid heating and cooling, can be secondary quenched [16]. The interaction between the mechanical force and grinding heat results in ultrafine grain sizes and phase-transition structures. Kang et al. [17] concluded that the increase in cutting depth and feed rate increased the degree of plastic deformation of the machined surface when grinding 17CrNiMo6 steel. Moreover, the increasing grinding temperature induced phase transformation, modified the microstructure and redistributed the microhardness of the machined surface layer. Lerra et al. [18] reported that, as the feed speed increased from 0.34 to 0.54 mm/rev, the typical martensite structure in the surface hardening depth was visible without remarkable thermal defects when 27MnCr5 gears were ground with an aluminum grinding wheel. However, when the silicon carbide abrasives were used, obvious grinding burns occurred on the tooth surface due to its lower thermal conductivity and the severe wear of the abrasive grits. 

Based on the comprehensive literature review, it was found that the grinding process significantly changes the surface roughness, morphology and the surface layer microstructure in the gear manufacturing process and further affected the gear performance. Therefore, as a typical difficult-to-cut material, it is necessary to study the performance of 20Cr2Ni4A during the form-grinding process. In this work, form grinding of 20Cr2Ni4A gears with different parameters was performed, and the effects of feed speed and cutting depth on the grinding temperature were analyzed. The distribution of tooth surface roughness and the formation mechanism of the machined surface morphology were studied comprehensively. Moreover, the characteristics of the microstructure in the tooth surface layer were investigated. The results of this study can mostly likely fill the gap in the research on form grinding 20Cr2Ni4A alloy steel and provide information for the form grinding of 20Cr2Ni4A gears in the automotive industry with high efficiency and quality.

## 2. Experimental Details

### 2.1. Materials

**Figure 1:**
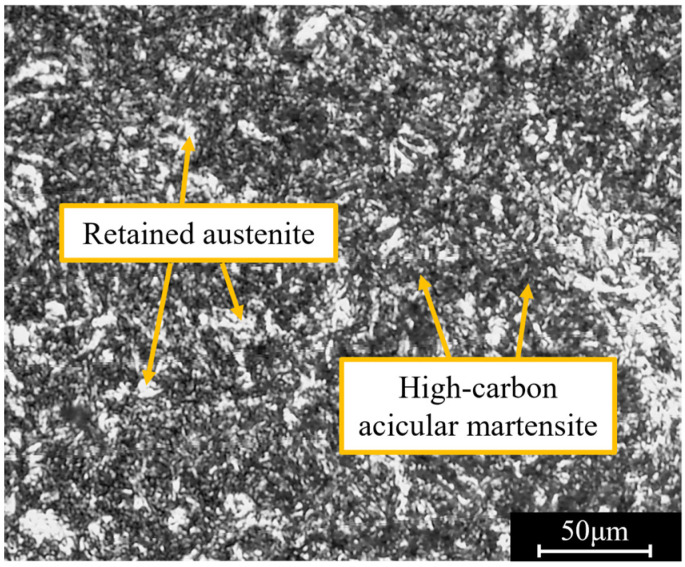
Microstructure in carburized layer of the 20Cr2Ni4A blank gear.

The chemical composition of a 20Cr2Ni4A steel gear is shown in Table 1. Before the experiment, the gear blanks were heat-treated through vacuum carburizing, high tempering, oil quenching and low tempering. Figure 1 shows the microstructure of the gear blanks, which was mainly composed of high-carbon acicular martensite, spheroidized carbide and retained austenite.

### 2.2. Experimental Procedure

**Figure 2:**
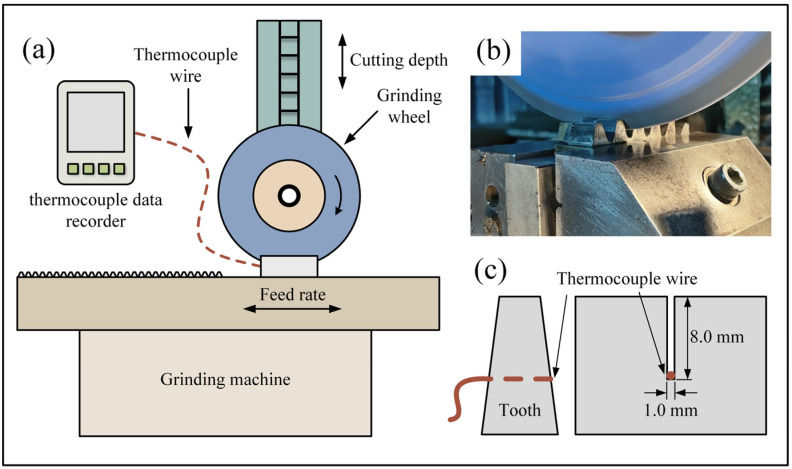
(**a**) Experiment setup, (**b**) grinding process and (**c**) installation of thermocouple.

**Figure 3:**
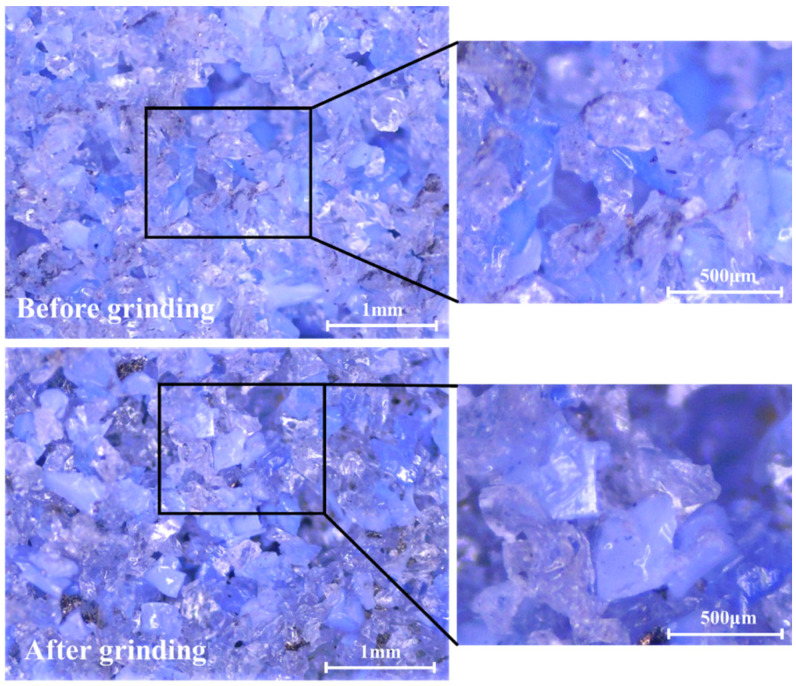
Surface topography of the alumina grinding wheel before and after grinding.

Figure 2 schematically shows the grinding system, including a high-precision grinding machine (HF-AS3060) with a constant spindle speed of 1450 rpm, an alumina forming grinding wheel with a diameter of 350 mm and an average grain size of 180 μm. Before the experiment, the grinding wheel was dressed to an involute profile using a diamond wheel dresser. Each of the workpieces had 6 teeth, which is shown in Figure 2b. As shown in Figure 3, the surface topography of the grinding wheel before and after the experiment was observed with a digital optical microscope. The sharp cutting edge of the abrasive grits was kept after grinding, and no visible wear marks were observed. Therefore, the effect of wheel wear on the experimental results can be neglected. Emulsion was used as a coolant during the form-grinding process. In order to investigate the influence of grinding parameters on tooth surface integrity, a full factorial experiment was designed. Feed speed (v_w_) and cutting depth (a_p_) with four levels were selected as the research factors by referring to on-site machining parameters. Detailed grinding parameters and corresponding levels are shown in Table 2.

### 2.3. Measurements

Grinding temperature was measured by K-type thermocouples and the data were recorded with a NAPU NP130T multichannel temperature recorder. The thermocouple was embedded in a groove with a depth of 8.0 mm and a width of 1.0 mm and was sealed with flame-retardant sealant. The grinding process with each group of parameters would continue until the temperature measurement results were stable and the highest temperature was recorded for each group. To reduce the effect of high-frequency errors and specific surface defects on the measurement results [19,20,21], tooth surface roughness Ra was measured with a contact stylus profiler (Time TR200) with an evaluation length of 4.0 mm and a sampling length of 0.8 mm. The tangential roughness was measured twice at both the tip and root of the tooth and averaged, respectively. Surface morphology was observed with a ZEISS Smartzoom 5 automated digital microscope. After the grinding process, specimens with a width of 8 mm were cut from the workpiece using electrical discharge wire cutting. The specimens were ground/polished and then etched with an alcohol solution containing 4 vol% of nitric acid. The microstructure was observed with an OLYMPUS-GX71 optical microscope and Coxem EM-30N scanning electron microscope.

## 3. Results and Discussion

### 3.1. Surface Morphology

**Figure 4:**
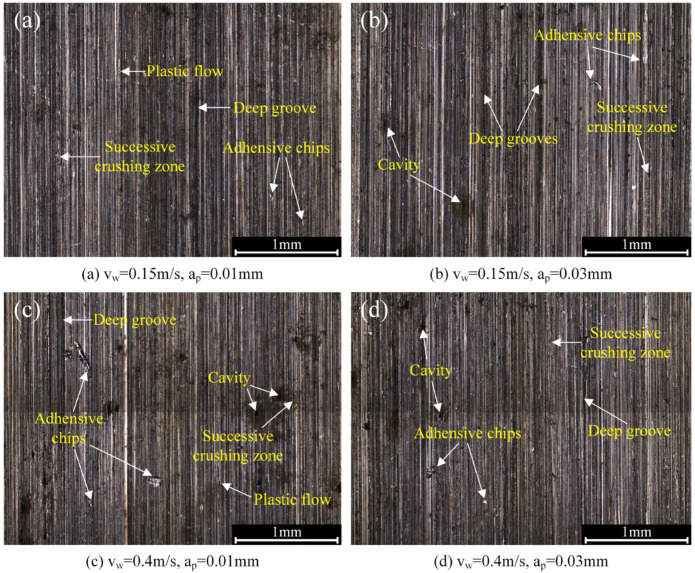
Tooth surface morphology after grinding with the parameter of (**a**) 0.15 m/s, 0.01 mm; (**b**) 0.15 m/s, ap = 0.03 mm; (**c**) 0.4 m/s, 0.01 mm; and (**d**) 0.4 m/s, 0.03 mm.

**Figure 5:**
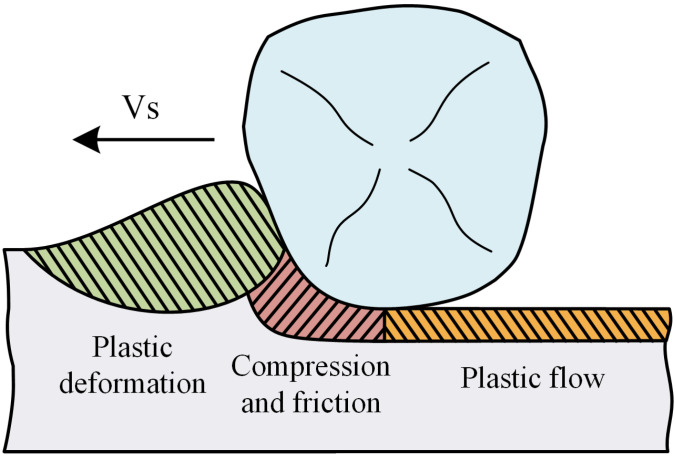
Schematic diagram of plastic flow formation.

Figure 4 shows the tooth surface morphology after the form-grinding process, which is a typical machining process of removing materials by multigrits and their interactions [22]. The morphology of the machined surface was mainly composed of cutting grooves. However, in some areas, the material was removed irregularly by the abrasive grits breaking or falling off from the grinding wheel, forming the successive crushing zone. Furthermore, plastic flow, adhesive chips, deep grooves and cavities were also observed. Plastic flow was a common machining defect caused by the grinding process. As shown in Figure 5, when the abrasive grits invaded a workpiece with a shallow depth, due to the ploughing effect, materials within the surface layer had a plastic upheaval in front of the abrasive grits. The shear stress was not high enough for a slip phenomenon to appear in the primary shear zone. Under the high compression stress and friction of the cutting edge, the material produced severe plastic deformations and flowed with the abrasive grits. Large positive rake angle and a blunt cutting edge intensified plastic flow. It was found that more plastic flow was introduced to the machined surface when ground with a relatively shallow cutting depth.

**Figure 6:**
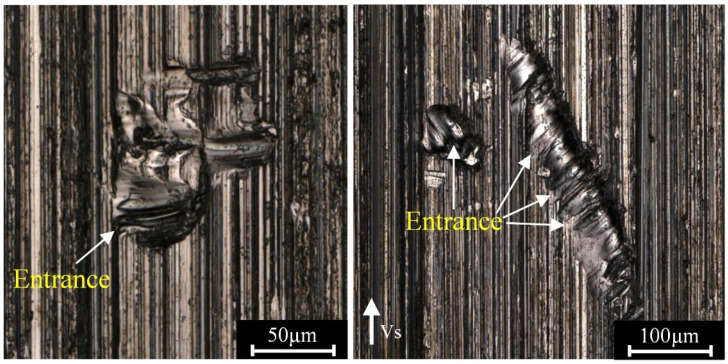
Typical surface morphology of adhesive chips.

Abrasive chips adhered to the machined surface were also regarded as a defect. During the form-grinding process, a part of the removed materials adhered to the wheel and then transferred back to the machined surface by friction-welding processes [23] when it contacted with the workpiece again, resulting in chip adhesion on the ground surface. As shown in Figure 6, grooves inconsistent with the cutting direction with some large adhesive chips were observed on the surface. The adhesive chips protruding from the machined surface might impact the abrasive grits. Some hard particles of broken or deboned abrasive grits could be embedded in the space between the abrasive grits and cut the material in an uncontrolled direction [24], generating unparallel grooves in the adhesive chips. Furthermore, the width of the groove entrance was significantly larger than that of the exit, indicating that the entrance was repeatedly impacted by the hard particles, and all the particles were released along the same path. Deep grooves in the machined surface were believed to be caused by the penetration of fallen or broken abrasive grits due to the interaction of the workpiece and the grinding wheel [25]. 

**Figure 7:**
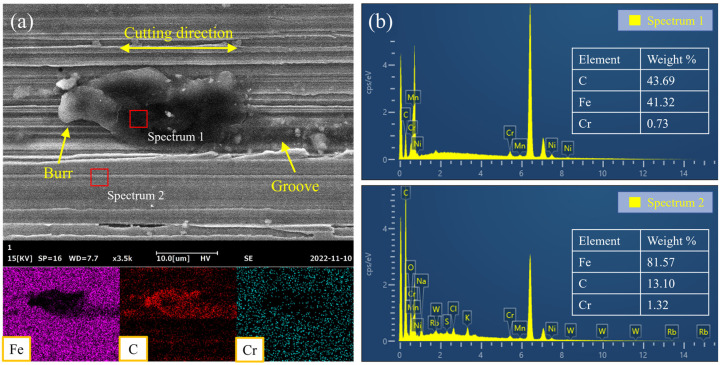
(**a**) Morphology and element map of the cavity and (**b**) corresponding EDS analysis for the form-ground samples.

Additionally, it was found that some cavities scattered over the machined surface. Figure 7 shows the morphology of the cavity and the distribution of surface elements. A burr was observed on one side of the cavity and a groove was located on the other side. It was clearly seen from the elemental maps that the concentration distribution of Fe and C highly coincided with the boundary of the cavity. The distribution of elements suggested that the cavities appearing on the machined surfaces were most likely the remnant of carbide particles. During the form-grinding process, the carbide particles pushed by abrasives could be considered substitutes for the cutting edge to remove material. Due to the double-pass grinding strategy used in this work, the carbide particles were pushed in two directions and produced burrs on both sides of the moving space. With the increase in the grinding depth, the exposed carbide particles increased until the compressive stress of the carbide particles on the metal matrix forereached the yield strength of the matrix, thereby crushing the bonding interface, breaking away and cutting the surface.

**Figure 8:**
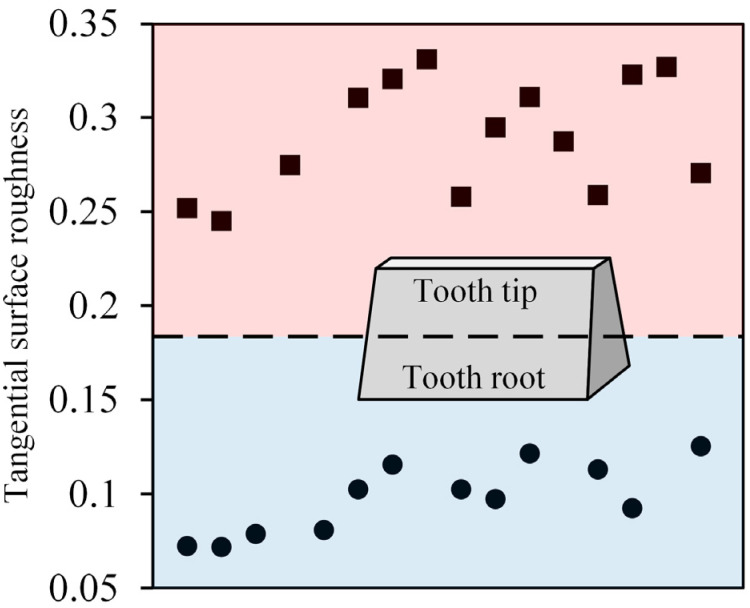
Distribution of tangential roughness values on the tooth surface.

**Figure 9:**
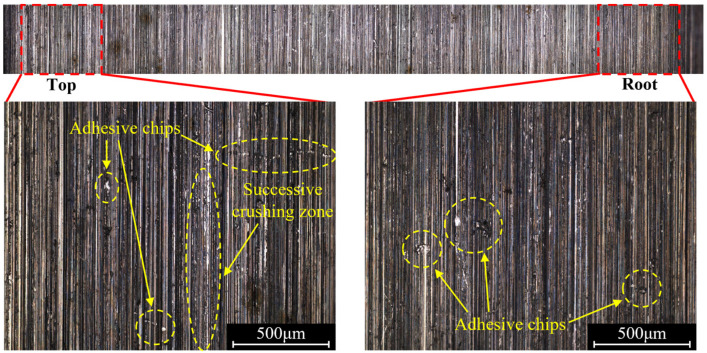
Surface morphology of tooth tip and tooth root.

**Figure 10:**
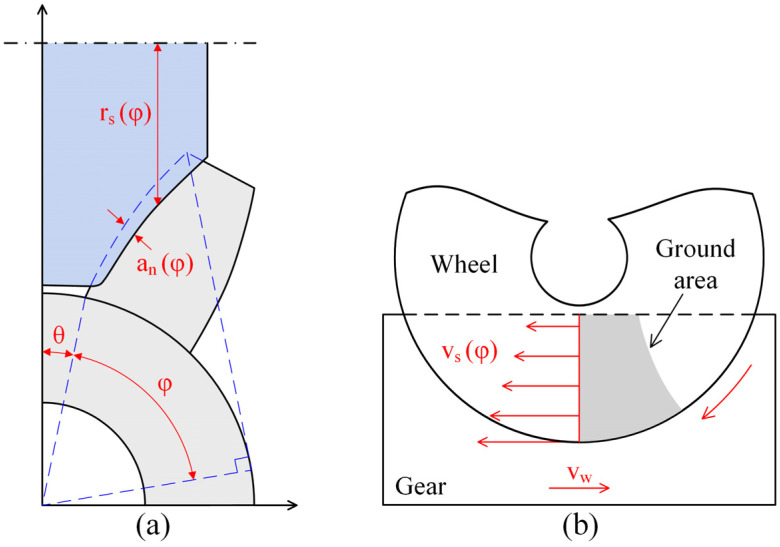
(**a**) Local wheel radius and cutting depth, and (**b**) local cutting depth and contact condition.

The distribution of tangential roughness on the tooth surface is shown in Figure 8. The average roughness of tooth tip was 0.28 μm, while the value at the root of the tooth was only 0.11 μm. Figure 9 shows the surface morphology from tooth tip to tooth root. Grinding marks on the tooth tip were more obvious, the successive crushing zone and a large number of adhesive chips were observed, while the machined surface of the tooth root was relatively smooth, and the size of adhesive chips was large. Different contact conditions between the grinding wheel and the workpiece were a possible reason for the transformation of the surface morphology. Figure 10 shows the cross section of the contact area between the grinding wheel and the tooth during the form grinding of the gears, where φ is the rolling angle of the involute tooth profile. The local diameter r_s_ (φ) of the grinding wheel at different contact positions was variable, resulting in the local cutting speed at the tooth root being higher than the one at the tooth tip. The maximum undeformed chip thickness of the abrasive grits decreased with the increase in cutting speed, which reduced the height of peaks and the depth of valleys on the machined surface. Therefore, the surface roughness decreased. In addition, the local cutting depths a_n_ (φ) at different contact positions were also different. According to the results from Jin et al. [26], a_n_ (φ) is equal to a_r_·cos (π/2 − φ − θ), where a_r_ is the radial cutting depth. The metal was cut with a higher local cutting depth on the tooth tip and deeper grinding marks were introduced, resulting in an increase in roughness. In addition, as shown in Figure 10b, the contact length between the tooth and the grinding wheel at the tooth tip was lower than the one at the tooth root during form grinding, which indicated that more abrasive grits participated in grinding simultaneously at the tooth root. The grinding marks of abrasive grits were more prone to interfere with each other, resulting in a decrease in the residual grinding height. Therefore, the surface of the tooth root was smooth. However, with the increase in the contact length between the grinding wheel and the tooth surface, it was difficult for the cutting fluid to cover the entire cutting area. As a result, more adhesive chips with large size occurred on the tooth root.

### 3.2. Microstructure Evolution

**Figure 11:**
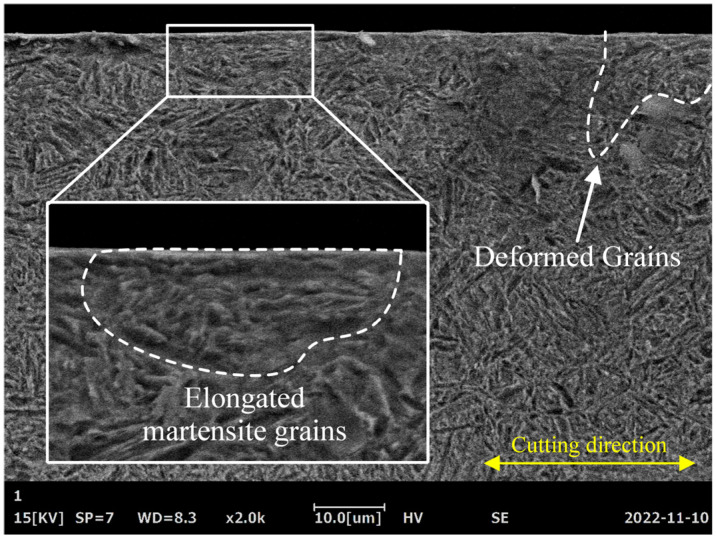
Typical morphology of the microstructure in the subsurface layer after grinding with a feed speed of 0.4 m/s and cutting depth of 0.03 mm.

Figure 11 shows the microstructure of the tooth surface layer parallel to the cutting direction after form grinding. After carburizing and quenching, the microstructure of the 20Cr2Ni4A steel gear was mainly composed of high carbon acicular martensite and residual austenite. Due to the effects of grinding heat and mechanical force in the form-grinding process, the grain size in the region adjacent to the ground surface was significantly reduced, and some grains were deformed along the cutting direction. During the grinding process, a large amount of heat generated in the cutting area flowed into the machined surface layer of the workpiece and was subsequently rapidly carried away by the coolant, causing grain refinement and quenching to harden the grain on the tooth surface. Meanwhile, under the action of mechanical force generated by the interaction between the form-grinding wheel and the workpiece, cold plastic deformation occurred on the machined surface layer, which caused shearing and slipping in the crystal, leading to dislocation multiplication. The interaction between dislocations, such as stacking and entanglement, increased the deformation resistance of the surface layer materials [17]. In addition, with the increase in plastic deformation, the substructure was refined, and the metallic structure was elongated, broken and fibrotic, which increased the deformation resistance and work hardening.

**Figure 12:**
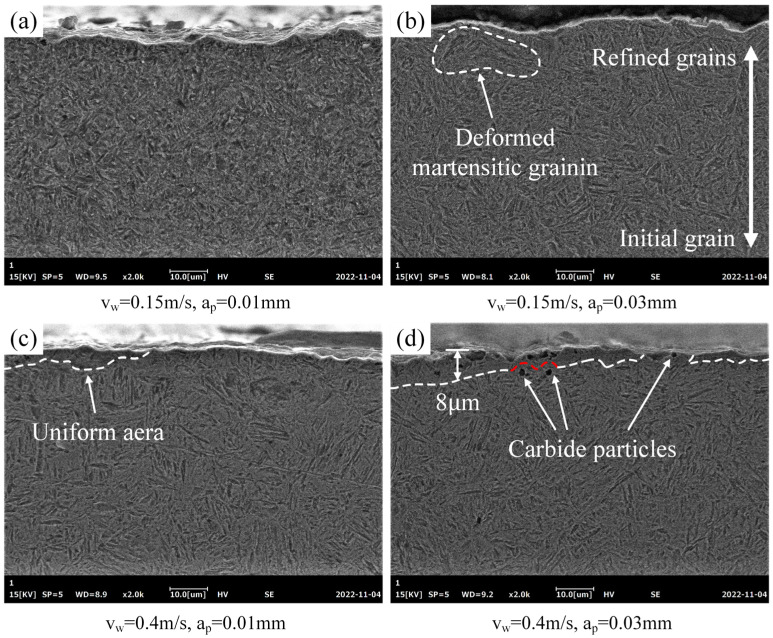
Microstructure of the machined surface layer under the parameters of (**a**) 0.15 m/s, 0.01 mm; (**b**) 0.15 m/s, ap = 0.03 mm; (**c**) 0.4 m/s, 0.01 mm; and (**d**) 0.4 m/s, 0.03 mm.

Figure 12 shows the microstructure of the tooth surface layer perpendicular to the cutting direction after grinding with different parameters. It can be seen from Figure 12a that, when the feed speed and cutting depth were 0.15 m/s and 0.01 mm, respectively, no obvious contrast between the microstructure in the surface layer and the deeper region was observed due to the low grinding force and temperature. As the cutting depth increased to 0.03 mm, the size of the martensite structure from the surface layer to the deeper region presented a trend from small to large. At the region close to the machined surface, the acicular martensite grains were more inclined to a horizontal arrangement. In addition, martensite grains adjacent to the machined surface were subjected to the normal pressure of the abrasive grits and deformed along the pile-up and sinking-in profile of the cross section of the machined surface.

As shown in Figure 12c,d, when the feed speed increased to 0.4 m/s, a uniform area with a clear boundary with the initial microstructure was observed on the surface layer, and the area was expanded when the cutting depth increased from 0.01 mm to 0.03 mm. In the uniform area, the original boundary of austenite and martensite phases were blurred, and only a few small martensite grains were found. The possible reason was that, under the action of mechanical force, the surface layer produced severe plastic deformation along the normal and tangential directions, leading to grain deformation and breakage. The original grain boundary was crushed and could not be found at this scale. Furthermore, it was found that the volume fraction of austenite in the machined surface layer significantly increased after the grinding process. When the grinding temperature reached the martensite–austenite transformation temperature in the surface layer, the martensite reversely transformed to austenite in a diffusive manner without incubation time. The nanometer size of the grains and the transfer of material such as carbon from the machining tool into the surface layer could hinder the martensitic transformation and stabilize the austenite phase [15,27]. It was noted that the maximum depth of the observed uniform area was about 8 μm, which indicated that, due to the short heat-source loading time and good cooling condition, a large amount of grinding heat could only reach to a limited depth in the surface layer. In addition, cavities due to carbide particles being removed during the polishing process were found in the surface layer. Due to their high hardness and stability, carbide particles could restrain the plastic deformation of materials by inhibiting sliding of the grain boundary during grinding [28]. 

**Figure 13:**
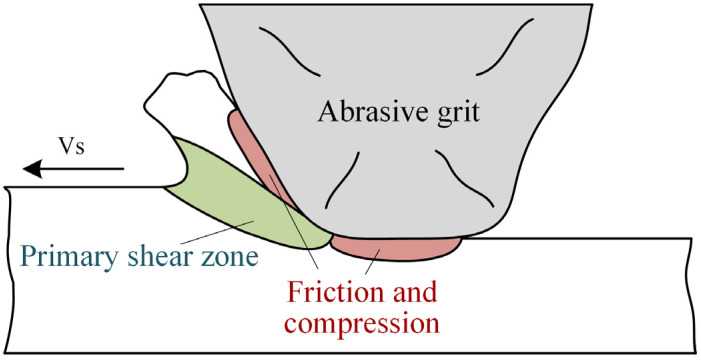
Schematic showing the main sources of grinding heat.

**Figure 14:**
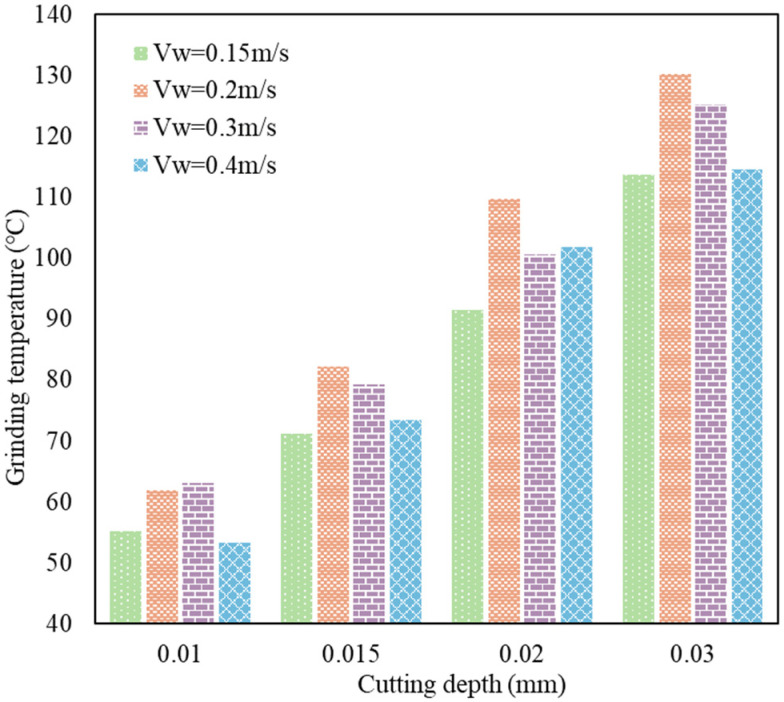
The influence of process parameters on grinding temperature.

During the form-grinding process, most of the grinding heat was provided by the elastic and plastic deformation of the workpiece, friction between the workpiece and abrasive grits, and friction between chips and abrasive grains, as shown in Figure 13. Then, the heat was transferred into the grinding chips, the cooling fluid, the grinding wheel and the workpiece, causing the temperature to rise in the grinding area. It can be seen from Figure 14 that the grinding temperature showed a significant positive correlation with cutting depth. With the increase in cutting depth, the contact arc length and maximum undeformed chip thickness increased, resulting in an increase in the energy released due to the severe elastic–plastic deformation of the workpiece material. Most of the energy was converted to heat and transferred into the workpiece, chips and fluid. In addition, the contact area between the grinding wheel and the workpiece increased and more frictional heat was produced. Moreover, when the cutting depth increased from 0.02 mm to 0.03 mm, the increase in grinding temperature slowed down. From the perspective of energy transmission, with the increase in cutting depth, the volume of the chips increased, and the specific surface area decreased. More heat flowed into the chips and was taken away from the workpiece, so the grinding temperature presented a relatively slow increase. Different from the consistent trend of variation in the grinding temperature with cutting depth, there was no linear correlation between the feed speed and grinding temperature. With the increase in feed speed, grinding temperature first increased to a maximum value and then decreased. The increase in feed speed enhanced the material removal in unit time and increased the contact area between the grinding wheel and the workpiece, resulting in an increase in the heat produced by material deformation and friction, as well as increased the heat source intensity in the grinding area. 

## 4. Conclusions

(1) Machining defects produced by the form-grinding process were determined to be successive crushing zones, plastic flow, adhesive chips, deep grooves and cavities. Adhesive chips could break abrasive grits and introduce more successive crushing zones and deep grooves to the machined surface. Cavities were left on the surface after carbide in the material was removed, which were most likely inevitable when using a relatively high level of grinding parameters.

(2) The contact condition between the grinding wheel and the tooth varied with the change in the involute rolling angle, resulting in the local cutting speed and contact length at the tooth root being higher than the one at the tooth tip, while the local cutting depth was smaller. Therefore, the machined surface of the tooth root was relatively smooth.

(3) The grinding force and heat introduced grain refinement and deformation of the microstructure in the machined surface layer, and they increased with the increase in cutting depth and feed speed. When the feed speed was 0.4 m/s, a uniform area with a blurry grain boundary was found, which might be attributed to mechanical deformation and an austenite phase reversal.

## Figures and Tables

**Table 1:** Chemical compositions of 20Cr2Ni4A steel (wt.%).

C	Si	Cr	Ni	Mn	Fe
0.21	0.25	1.45	3.55	0.48	Bal.

**Table 2:** Details of the process parameters in the gear form-grinding experiments.

Factors	Level 1	Level 2	Level 3	Level 4
Feed speed (v_w_, m/s)	0.15	0.2	0.3	0.4
Cutting depth (a_p_, mm)	0.01	0.015	0.02	0.03

## Data Availability

The data that support the findings of this study are available from the corresponding author upon reasonable request.

## References

[1] Wei J., Pan Z., Lin X., Qin D., Zhang A., Shi L. (2019). Copula-function-based analysis model and dynamic reliability of a gear transmission system considering failure correlations. Fatigue Fract. Eng. Mater..

[2] Wang Y., Xing Z., Huang Y., Guo W., Kang J., Wang H., Zhang Z. (2021). Effect of pulse magnetic field treatment on the hardness of 20Cr2Ni4A steel. J. Magn. Magn. Mater..

[3] Jiang X., Liu K., Yan Y., Li M., Gong P., He H. (2022). Grinding temperature and surface integrity of quenched automotive transmission gear during the form grinding process. Materials.

[4] Fernandes C.M., Martins R.C., Seabra J.H. (2016). Coefficient of friction equation for gears based on a modified Hersey parameter. Tribol. Int..

[5] Zhou W., Tang J., He Y., Liao D. (2015). Associated rules between microstructure characterization parameters and contact characteristic parameters of two cylinders. J. Cent. South Univ..

[6] Yi J., Jin T., Zhou W., Deng Z. (2020). Theoretical and experimental analysis of temperature distribution during full tooth groove form grinding. J. Manuf. Process..

[7] Su J., Ke Q., Deng X., Ren X. (2018). Numerical simulation and experimental analysis of temperature field of gear form grinding. Int. J. Adv. Manuf. Technol..

[8] Yi J., Jin T., Deng Z. (2019). The temperature field study on the three-dimensional surface moving heat source model in involute gear form grinding. Int. J. Adv. Manuf. Technol..

[9] Öpöz T.T., Chen X. (2012). Experimental investigation of material removal mechanism in single grit grinding. Int. J. Mach. Tool. Manuf..

[10] Yang L., Wang L., Liu Q., Tian X. (2017). Grinding performance of a new micro-crystalline corundum wheel when form-grinding automobile gears. Int. J. Adv. Manuf. Technol..

[11] Zhou K., Ding H., Wang R., Yang J., Guo J., Liu Q., Wang W. (2020). Experimental investigation on material removal mechanism during rail grinding at different forward speeds. Tribol. Int..

[12] Ma X., Cai Z., Yao B., Chen G., Cai S., Liu W.S. (2022). Prediction model for surface generation mechanism and roughness in face gear grinding. Int. J. Adv. Manuf. Technol..

[13] Zhou W., Tang J., Shao W. (2020). Study on surface generation mechanism and roughness distribution in gear profile grinding. Int. J. Mech. Sci..

[14] Yang M., Chen J., Wu C., Xie G., Jiang X., Li M., He H. (2022). Residual stress evolution and surface morphology in grinding of hardened steel following carburizing and quenching processes. Materialwiss. Werkst..

[15] Le Nué C., Deschamps A., Danoix F., de Geuser F., Renou G., Verdier M., Billot T., Binot N., Dides C. (2021). Influence of grinding and shot-peening on the near-surface microstructure of a maraging stainless steel. Materialia.

[16] Wang L., Tang X., Wang L., Yang N., Chen X., Li P., Liu G., Liu G. (2019). Mechanism of grinding-induced burns and cracks in 20CrMnTi steel gear. Mater. Manuf. Process..

[17] Kang B., Ma H., Li J., Xu B. (2020). Effect of grinding parameters on surface quality, microstructure and rolling contact fatigue behaviors of gear steel for vacuum pump. Vacuum.

[18] Lerra F., Grippo F., Landi E., Fortunato A. (2022). Surface integrity evaluation within dry grinding process on automotive gears. Clean. Eng. Technol..

[19] Han H. (2015). Uncertainty in measurement of surface topography. Surf. Topogr-Metrol..

[20] Podulka P. (2022). Selection of Methods of Surface Texture Characterisation for Reduction of the Frequency-Based Errors in the Measurement and Data Analysis Processes. Sensors.

[21] Giusca C.L., Claverley J.D., Sun W., Leach R.K., Helmli F., Chavigner M.P.J. (2014). Practical estimation of measurement noise and flatness deviation on focus variation microscopes. CIRP Ann. Technol..

[22] Chen X., Öpöz T.T., Oluwajobi A. (2017). Analysis of grinding surface creation by single-grit approach. J. Manuf. Sci. Eng..

[23] Zhou N., Peng R., Pettersson R. (2016). Surface integrity of 2304 duplex stainless steel after different grinding operations. J. Mater. Process. Technol..

[24] Miao Q., Ding W., Gu Y., Xu J. (2019). Comparative investigation on wear behavior of brown alumina and microcrystalline alumina abrasive wheels during creep feed grinding of different nickel-based superalloys. Wear.

[25] Zhao Q., Guo B. (2015). Ultra-Precision grinding of optical glasses using mono-layer nickel electroplated coarse-grained diamond wheels. Part 2: Investigation of profile and surface grinding. Precis. Eng..

[26] Jin T., Yi J., Cai R. (2016). Investigation on the Grinding Force, Power and Heat Flux Distributions Along the Tooth Profile in Form Grinding of Gears. Proceedings of the International Manufacturing Science and Engineering Conference.

[27] Lee S.J., Park Y.M., Lee Y.K. (2009). Reverse transformation mechanism of martensite to austenite in a metastable austenitic alloy. Mater. Sci. Eng. A-Struct..

[28] Wang Z., Yan Y., Xing L., Su Y., Qiao L. (2017). The role of hard phase carbides in tribocorrosion processes for a Co-based biomedical alloy. Tribol. Int..

